# *Npas3* regulates stemness maintenance of radial glial cells and neuronal migration in the developing mouse cerebral cortex

**DOI:** 10.3389/fncel.2022.865681

**Published:** 2022-10-13

**Authors:** Ji-Wei Liu, Han Li, Yang Zhang

**Affiliations:** Shanghai Mental Health Center, Shanghai Jiao Tong University School of Medicine, Shanghai, China

**Keywords:** NPAS3, radial glial cells, stemness maintenance, neuronal migration, cerebral cortex

## Abstract

The neuronal PAS domain 3 (NPAS3) is a member of the basic helix-loop-helix (bHLH) PAS family of transcription factors and is implicated in psychiatric and neurodevelopmental disorders. *NPAS3* is robustly expressed in the cortical ventricle zone (VZ), a transient proliferative zone containing progenitor cells, mainly radial glial cells, destined to give rise to cortical excitatory neurons. However, the role of *NPAS3* in corticogenesis remains largely unknown. In this study, we knocked down *Npas3* expression in the neural progenitor cells residing in the cortical VZ to investigate the role of *Npas3* in cerebral cortical development in mice. We demonstrated that *Npas3* knockdown profoundly impaired neuronal radial migration and changed the laminar cell fate of the cells detained in the deep cortical layers. Furthermore, the downregulation of *Npas3* led to the stemness maintenance of radial glial cells and increased the proliferation rate of neural progenitor cells residing in the VZ/subventricular zone (SVZ). These findings underline the function of *Npas3* in the development of the cerebral cortex and may shed light on the etiology of *NPAS3*-related disorders.

## Introduction

Neuronal PAS domain 3 (NPAS3) is a member of basic helix-loop-helix (bHLH) PAS domain transcription factors that are highly enriched in the developing and adult brain (Brunskill et al., 1999; Kamnasaran et al., 2003; Gould and Kamnasaran, 2011; Kamm et al., 2013). The *NPAS3* gene contains 12 exons encoding three functional domains (Kamnasaran et al., 2003): a bHLH domain responsible for binding to the promoters of target genes (Kewley et al., 2004), paired Per-Arnt-Sim (PAS) domains sensing subtle environmental changes to alter the affinity for heterodimeric partner binding (Kewley et al., 2004; McIntosh et al., 2010), and a functional transcription activation domain (TAD) responsible for modulation of target gene expression (Luoma and Berry, 2018). NPAS3 is involved in multiple biological functions, such as cell differentiation, circadian rhythms, and nervous system development (Brunskill et al., 1999; Kamnasaran et al., 2003; Gould and Kamnasaran, 2011; Kamm et al., 2013). Disruption of the human *NPAS3* gene by the reciprocal translocation at 14q13 has been first reported in a family with intellectual disability and schizotypal features (Kamnasaran et al., 2003; Pickard et al., 2005), indicating that disruption of the *NPAS3* gene may contribute to the origin and development of psychiatric disorders. Subsequent studies have further linked *NPAS3* aberrations to multiple human psychiatric and neurodevelopmental disorders, including schizophrenia (Pickard et al., 2005, 2006, 2009; Huang et al., 2010; Macintyre et al., 2010; Yu et al., 2014; Gonzalez-Penas et al., 2015), bipolar disorder (Pickard et al., 2009; Huang et al., 2010; Nurnberger et al., 2014), major depression (Huang et al., 2010), attention-deficit/hyperactivity disorder (Weber et al., 2011), and intellectual disability (Pickard et al., 2005; Visser et al., 2010; Phelps et al., 2017). In support of the implication of *NPAS3* in neuropsychiatric and neurodevelopmental disorders, mouse knockout models of *Npas3* display a range of behavioral phenotypes including locomotor hyperactivity, subtle gait defects, memory problems, and impairment of prepulse inhibition (Erbel-Sieler et al., 2004; Brunskill et al., 2005; Pieper et al., 2005). *Npas3* knockout mice exhibit deficits in neurodevelopment resulting in neuroanatomical alterations such as enlarged ventricles, reduced corpus callosum, and hippocampal volume (Brunskill et al., 2005). In addition, reduced adult neurogenesis in the dentate gyrus of the hippocampus (Pieper et al., 2005) and decreased number of neocortical interneurons are also observed following *Npas3* ablation (Stanco et al., 2014). These results suggest that *Npas3* is involved in processes that are essential for normal brain development.

During the prenatal development of mouse and human nervous systems, *Npas3* is vigorously expressed in the cortical ventricle zone (VZ) (Gould and Kamnasaran, 2011; Stanco et al., 2014), a transient primary proliferative zone containing neural stem cells (principally radial glia cells) (Martinez-Cerdeno and Noctor, 2018), raising the possibility that *Npas3* is also implicated in the neurogenesis and development of the cerebral cortex. In the present study, short hairpin RNAs (shRNAs) delivered by *in utero* electroporation were used to knockdown the expression of *Npas3* in the neural progenitor cells residing in VZ and their progeny at embryonic day 14.5 (E14.5). We demonstrated that the downregulation of *Npas3* led to neuronal radial migration defects and disturbed the laminar identity of cells detained in deep cortical layers. We also revealed that knockdown of *Npas3* resulted in the stemness maintenance of radial glial cells and increased the proliferation rate of neural progenitors in the VZ/subventricular zone (SVZ). Our findings provide insights into the function of *Npas3* in cerebral cortical development and the pathogenesis of *Npas3*-related neuropsychiatric disorders.

## Materials and methods

### Plasmids

To generate a GFP-tagged full-length *Npas3* (*Npas3*-*GFP*), mouse *Npas3* was amplified from a cDNA library of mouse cortex with oligonucleotide primers (5′-TCGAGCTCAAGCTTCGAATTCGCCACCATGGGGAGGGCCGGCGCCGCGG-3′ and 5′-CTCACCATGGTGGCGACCGGTAAGTCCTCCTTGCGCTCCAGAGTC-3′) and cloned into the pCAG-EGFP-N1 vector, which was constructed by replacing the human cytomegalovirus (CMV) promoter with a CMV early enhancer/chicken-actin (CAG) promoter of pEGFP-N1 (Clontech). Two oligonucleotides targeting the open reading frame (ORF) region of mouse *Npas3* were designed using BLOCK-iT™ RNAi Designer (Invitrogen) and inserted into the pSuper-basic vector (OligoEngine, VEC-PBS-0002) through *Bg*lII and *Hind*III restriction sites. The 19-nucleotide target sequences are as follows: 5′-GCACTGAGAAAGGAGAAAT-3′ (shRNA #1) and 5′-GGAGTCCACATCAAGTCAT-3′ (shRNA #2).

### Animals

C57BL/6J mice were purchased from Charles River Laboratories (Beijing Vital River Laboratory Animal Technology) and housed in 12 h light/dark cycles in a temperature-controlled room and given *ad libitum* access to food and water. All experimental procedures were in accordance with all institutional guidelines and ethics and were approved by the Institutional Animal Care and Use Committee of Shanghai Mental Health Center at Shanghai Jiao Tong University School of Medicine.

### Real-time PCR

Total RNA was prepared from the cerebral cortex of C57BL/6J mouse with TRIzol Reagent (Invitrogen). RNA was reversely transcribed by M-MLV transcriptase (BIO-RAD, 1708891) and amplified using SYBR Green Master Premix (Roche, 04887352001) following the manufacturer's protocol. For the amplification of mouse *Npas3* cDNA, a pair of primers, 5′-TACCAGCAGCGAATAACT-3′ and 5′-TTCACAGTAGATTCCGTCATA-3′ were used. For mouse glyceraldehyde-3-phosphate dehydrogenase (GAPDH), the primers were 5′-TGGCAAAGTGGAGATTGT-3′ and 5′-GTGGAGTCATACTGGAACA-3′.

### Cell culture and transfection

HEK 293T cells were cultured in Dulbecco's modified Eagle's medium (DMEM, Invitrogen) supplemented with 10% fetal bovine serum (FBS, Thermo Fisher Scientific, 10099141C). *Npas3*-*GFP* overexpression plasmid together with shRNAs or pSuper-basic vector at a ratio of 1:3 were transfected into HEK 293T cells using Lipofectamine 2000 transfection reagent (Invitrogen, 11668) according to the manufacturer's protocol. Cells were incubated at 37°C with 5% CO_2_ and 100% humidity and harvested for Western blot 48 h after transfection.

### Western blot

HEK 293T cells were lysed in lysis buffer (140 mM NaCl, 5 mM EDTA, 10 Mm Tris–HCl, and 0.2% Triton X-100) supplemented with 1 × protease inhibitor cocktail. After centrifugation, the supernatant was electrophoresed on 9% sodium dodecyl sulfate polyacrylamide gel and transferred to polyvinylidene fluoride membranes (Millipore). Membranes were blocked with 5% skimmed milk in TBST buffer (50 mM Tris–HCl, 140 mM NaCl, pH 7.4, with 0.05% Tween-20) at room temperature for 1 h, followed by incubation with rabbit anti-GFP antibody (1:2,000, Invitrogen, A11122) and horseradish peroxidase (HRP)-conjugated anti-GAPDH antibody (1:8,000, Kangchen, KC-5G4) at 4°C overnight. The membranes were rinsed three times the next day before incubating with HRP-conjugated anti-rabbit secondary antibody for 3 h at room temperature. Bands were visualized by Pro-Light HRP Chemiluminescent Kit (TIANGEN, PA112).

### *In utero* electroporation

*In utero* electroporation was performed as previously described (Liu et al., 2010). Briefly, pregnant C57BL/6J mice at E14.5 were anesthetized with pentobarbital sodium (70 mg/kg, i.p.) and the uterine horns were exposed. One microliter DNA solution consists of the pCAG-EGFP-N1 vector (0.3 μg/μl) and *Npas3* shRNAs (1 μg/μl) together with 0.01% fast green dye (Sigma-Aldrich, F7258) was delivered to the lateral ventricle using a glass micropipette. The electroporation was applied at 30 V (with 50 ms duration, 1 s interval, a total of five pulses) using an ECM830 electroporator (BTX Molecular Delivery Systems).

### Immunohistochemistry

Mice were sacrificed under anesthesia with pentobarbital sodium (70 mg/kg, i.p.) and fixed by intracardiac perfusion using 4% paraformaldehyde in 0.1 M phosphate-buffered saline (PBS) followed by postfixation in the same solution overnight at 4°C. For embryos at E16.5, brains were dissected out directly and fixed in 4% PFA overnight at 4°C. Fixed brains were dehydrated in 30% sucrose in 0.1 M PBS overnight before being embedded in OCT and cut into 50 μm coronal sections with a cryostat (Leica, CM1900). Immunohistochemistry was processed following the standard protocol. Sections were blocked with 5% BSA and 0.3% Triton X-100 in PBS for 1 h at room temperature, followed by incubation with primary antibodies at 4°C overnight. The sections were washed with PBS the next day and incubated with appropriate fluorescent secondary antibodies for 3 h at room temperature. Primary antibodies against the following proteins were used: rabbit anti-GFP (Invitrogen, a11122), chicken anti-GFP (Abcam, ab13970), rabbit anti-Cux1 (Santa Cruz, sc13024), rabbit anti-Cux1 (Proteintech, 11733-1-AP), mouse anti-Sox2 (Santa Cruz Biotechnology, sc-365823), rabbit anti-Pax6 (Cell Signaling Technology, 60433), rat anti-Ctip2 (Abcam, ab18465), rabbit anti-NeuoroD2 (Abcam, ab104430), rabbit anti-BLBP (Abcam, ab32423), rat anti-BrdU, (Abcam, ab6326), rabbit anti-Ki67 (Thermo Fisher Scientific, MA5-14520), and mouse anti-β-tubulin III (Tuj1, Sigma-Aldrich, MAB1637).

### BrdU labeling and detection

Bromo-2-deoxyuridine (BrdU) labeling assay was performed as previously described with modifications (Wojtowicz and Kee, 2006). Pregnant females at E16.5 were injected intraperitoneally with thymidine analog 5-bromo-2-deoxyuridine (BrdU, Sigma-Aldrich, B5002) at a concentration of 200 mg/kg body weight, 2 days after electroporation. Embryo brains were harvested 2 h later and fixed in 4% PFA overnight, followed by dehydration in 30% sucrose. Cryosections were incubated with 2 M HCl for 15 min at 37°C to denature the double-stranded DNA and allow access for the anti-BrdU antibody, then rinsed in PBS 3 times to neutralize the acid. After denaturation, sections were processed to immunostaining with anti-BrdU antibody or anti-Ki67 antibody.

### Axon length calculation

Brain slices from P7 mice were stained with Hoechst and GFP. Axon length was calculated as previously described (Nakashima et al., 2018).

### Image acquisition and statistics

Confocal images were captured using Nikon A1R. The brightness of images was adjusted in Adobe Photoshop CS4 and assembled in Adobe Illustrator CS4. Results are represented as mean ± standard error of the mean (SEM). One-way and two-way ANOVA tests were used for statistical analysis, and *p*-values considered significant were indicated by asterisks as follows: ^*^p < 0.05, ^**^p < 0.01, ^***^p < 0.001, ^****^p < 0.0001.

## Results

### Expression pattern of *Npas3* in the developing mouse cerebral cortex

**Figure 1:**
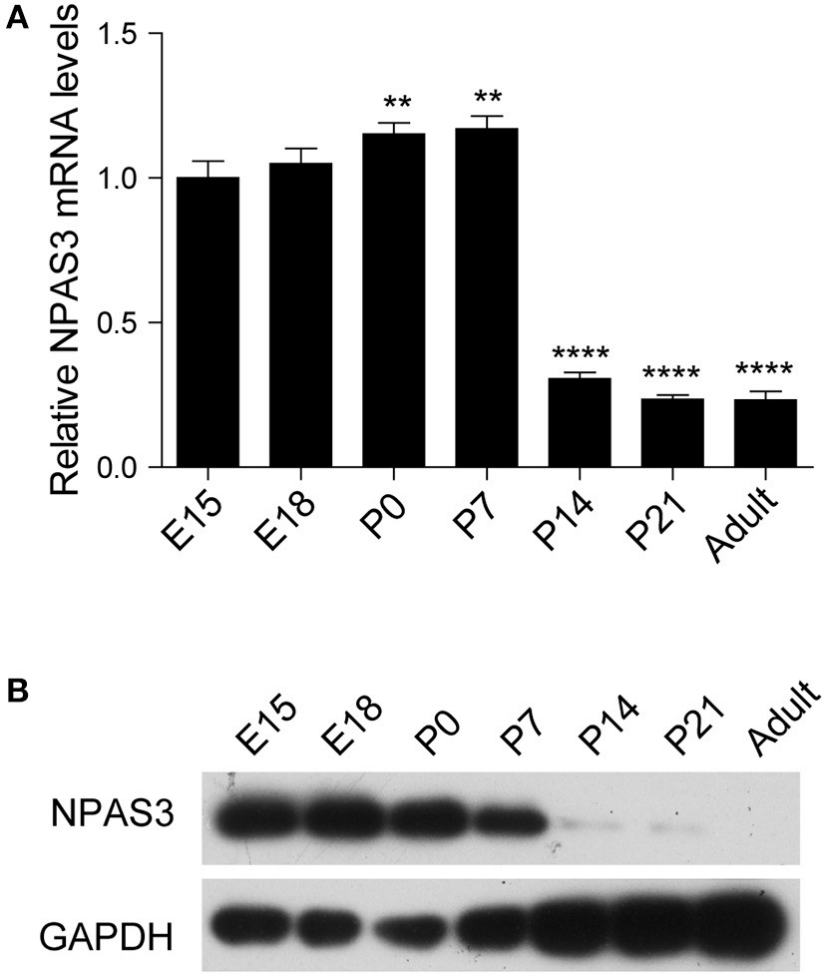
The expression of *Npas3* in the developing mouse cerebral cortex. **(A)** Quantification for *Npas3* mRNA levels in the developing cerebral cortex at the indicated stages obtained by RT-PCR. Data are presented as mean ± SEM (*n* = 3). One-way ANOVA test, **p < 0.01, ****p < 0.0001. **(B)** Western blot analysis of NPAS3 protein levels in the developing cerebral cortex at the indicated stages. GAPDH was used as a loading control.

To begin our studies on the role of *Npas3* in the development of the cerebral cortex, we first examined the temporal expression profile of *Npas3* in the developing mouse cerebral cortex. Both *Npas3* mRNA and protein levels were expressed at high levels during prenatal and early postnatal stages (from E15 to postnatal day 7 {P7}), then its expression declined significantly from P14 to adult (Figures 1A,B). Single-cell RNA-seq data reported by Loo et al. (2019) revealed that *Npas3* was expressed in the radial glia cells at E14.5 and interneurons at P0 (Supplementary Figure S1). These results suggest a possible role of *Npas3* in the prenatal and early postnatal development of the cerebral cortex.

### Impaired neuronal radial migration of *Npas3* knockdown cells

**Figure 2:**
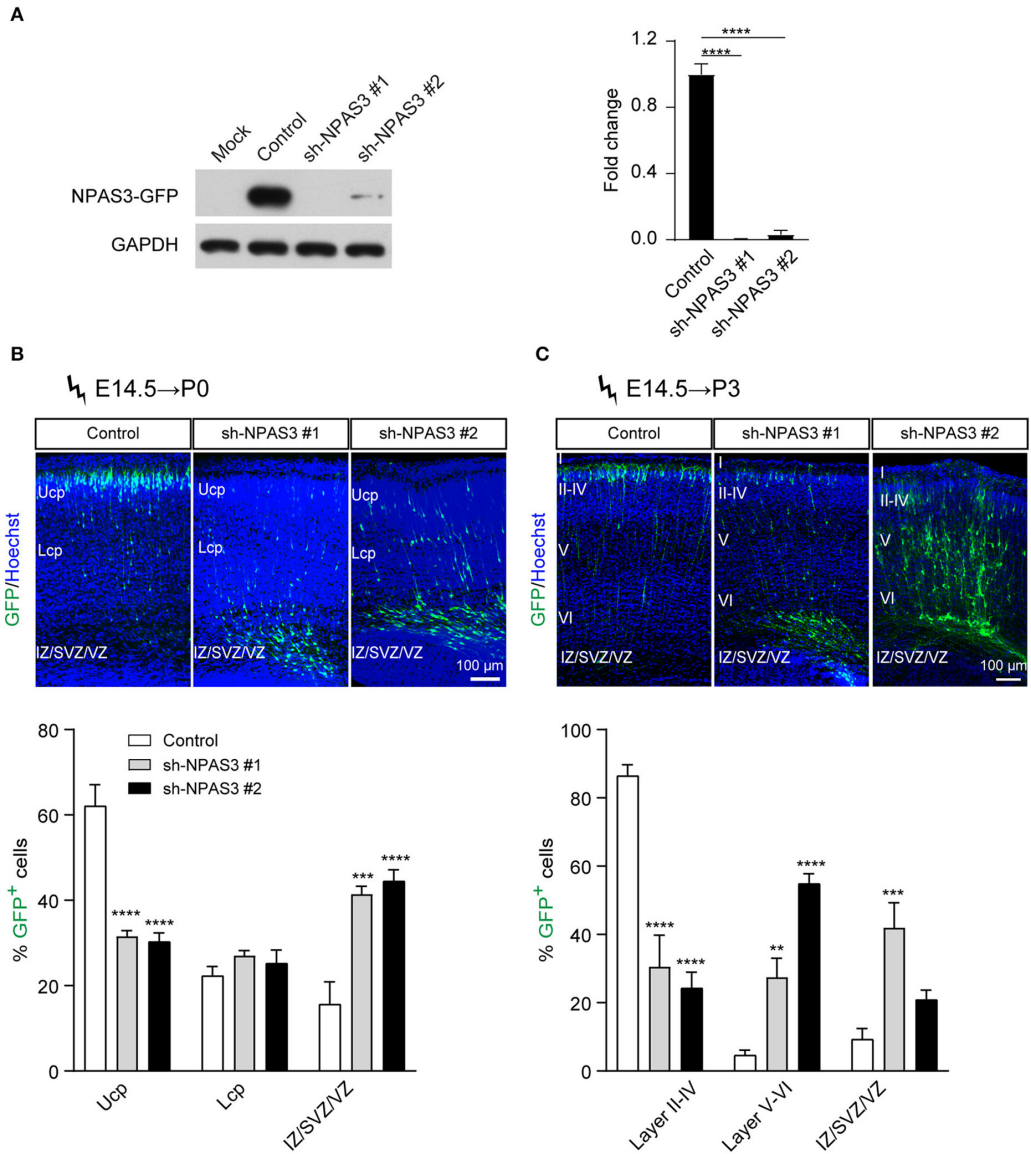
*Npas3* downregulation impaired neuronal radial migration *in vivo*. **(A)** Non-transfected HEK293T cells (mock) or cells transfected with an expression plasmid encoding mouse NPAS3-GFP protein together with *Npas3* shRNAs or control pSuper-basic plasmid were lysed 48 h after transfection and processed to Western blot analysis with anti-GFP and anti-GAPDH antibodies. Quantification of the relative protein levels is shown in the right panel. Data are presented as mean ± SEM (*n* = 3). One-way ANOVA test, ****p < 0.0001. GAPDH was used as a loading control. **(B)** and **(C)** Positioning defects of *Npas3*-knockdown cells. E14.5 mouse embryos electroporated with *Npas3* shRNAs or control pSuper-basic plasmid together with a GFP expression plasmid were allowed to develop until P0 **(B)** and P3 **(C)**. Electroporated cells are in green, and nuclei are in blue. Quantification of cell position is shown on the right panel. Data are presented as mean ± SEM (*n* = 3–5). Two-way ANOVA test, **p < 0.01, ***p < 0.001, ****p < 0.0001. Ucp, upper cortical plate; Lcp, lower cortical plate; IZ, intermediate zone; SVZ, subventricular zone; Cortical layers are labeled I–VI. Scale bar, 100 μm.

To investigate the role of *Npas3* in cerebral cortical development, we generated two shRNAs targeting the coding sequence of the *Npas3* gene for gene silencing. The shRNAs were driven by the U6 promoter in the pSuper-basic vector (OligoEngine), and their ability to knock down *Npas3* was tested in a transient transfection assay, in which C-terminally GFP-tagged *Npas3* was cotransfected with control pSuper-basic or shRNA constructs into HEK 293T cells. Western blot analysis 48 h after the transfection revealed that both shRNA#1 and shRNA #2 efficiently knocked down the expression of exogenous *Npas3-GFP* in HEK 293T cells, with shRNA #1 exhibiting a higher efficiency (Figure 2A).

We next used the method of *in utero* electroporation (Taniguchi et al., 2012) of shRNA constructs into the developing cortex to knockdown the expression of *Npas3* in a subpopulation of progenitors residing in cortical VZ and their progeny. A marker pCAG-EGFP plasmid for readily identifying electroporated cells (Bai et al., 2003) was coinjected with *Npas3* shRNA constructs into the lateral ventricle of E14.5 mouse embryos. After allowing the *in vivo* development, brains were harvested at P0 and P3 and cut at the level of the somatosensory cortex. Coronal sectioning with native GFP fluorescence of these brains revealed that most control cells transfected with pSuper-basic vector had reached the upper cortical plate (Ucp) at P0, while the majority of *Npas3* knockdown cells were stalled in the intermediate zone (IZ) and VZ/SVZ (Figure 2B). Similar results were observed at P3 (Figure 2C). We further assessed the callosal axon growth at P7 and found that the length of axons in the corpus callosum was comparable between groups (Supplementary Figure S2).

### Change of laminar identity in *Npas3* knockdown cells that detained in the deep cortical layers

**Figure 3:**
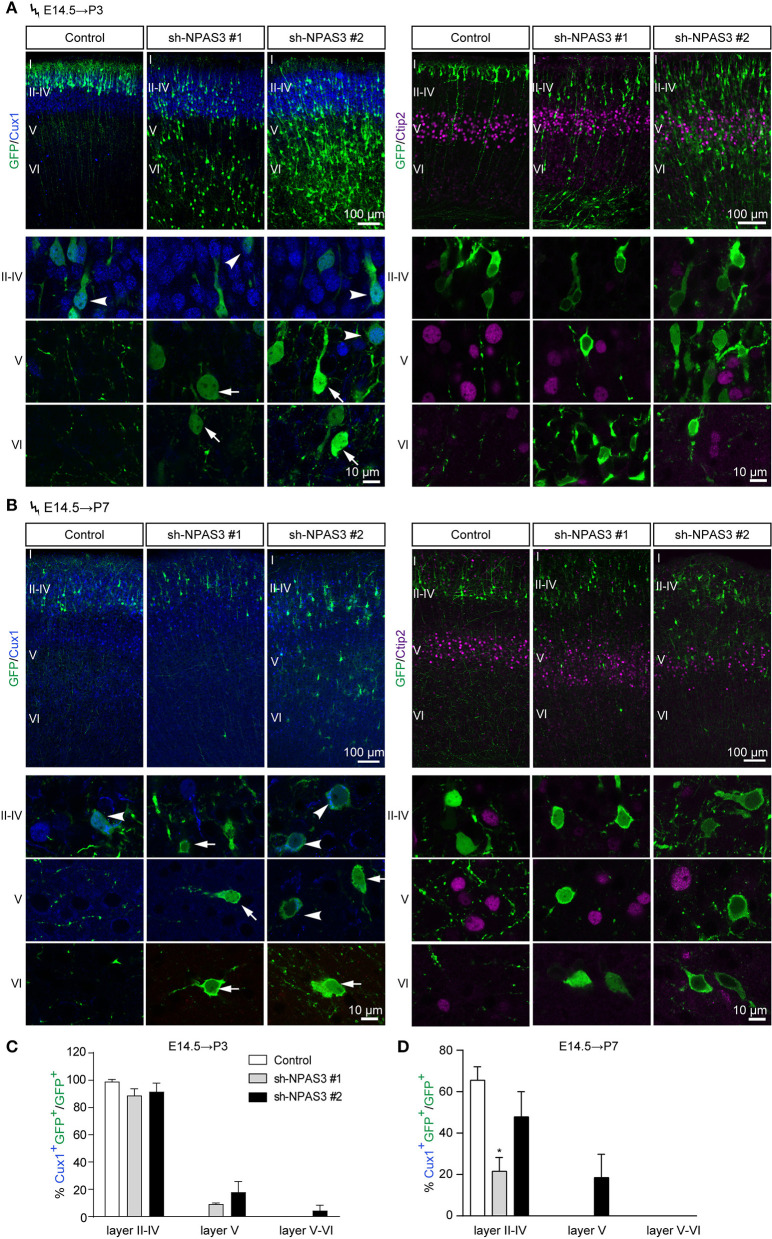
*Npas3* knockdown affected the laminar fate of cells detained in deep cortical layers. **(A,B)** Coronal sections of P3 **(A)** and P7 **(B)** brains electroporated at E14.5 were subjected to immunostaining with the anti-GFP antibody together with anti-Cux1 or anti-Ctip2 antibodies. Representative images with higher magnifications of *Npas3* knockdown cells in layers II–IV, V and VI are shown in the lower panels. Note that most *Npas3* knockdown cells in upper layers II–IV are Cux1 positive (arrowheads), while the majority of those in deep layers V and VI are Cux1 negative (arrows). Electroporated cells are in green, and nuclei are in blue. Cortical layers are labeled I–VI. Quantification of Cux1 positive cells from **(A)** and **(B)** is shown in **(C)** and **(D)** as % of total electroporated cells, ±SEM (*n* = 3–5). One-way ANOVA test, *p < 0.05.

Electroporation in the cortical progenitors of the VZ zone at E14.5 results in the targeting of cells that mainly differentiate into pyramidal neurons destined for layers II-IV (Fan et al., 2008). To examine whether the laminar identity of *Npas3* deficiency cells that detained in deep layers was changed, we conducted *in utero* electroporation at E14.5 and harvested the brains at P3 and P7. Immunostaining of GFP together with Cux1, a specific marker for upper layers II-IV neurons (Nieto et al., 2004), revealed that the majority of control and *Npas3* knockdown cells that had reached layers II–IV at P3 were Cux1 positive. In contrast, only a few of the *Npas3* knockdown cells that stalled in the deep cortical layers were Cux1 positive (Figures 3A–D). Surprisingly, these cells were not Ctip2 [a maker of layer V neurons (Arlotta et al., 2005)] positive either (Figures 3A,B). These results suggest that the laminar commitment of the *Npas3* knockdown cells that detained in the deep cortical layers was disrupted, which may consequentially lead to neuronal migration defects.

### *Npas3* knockdown promotes the stemness maintenance of the radial glial cells

**Figure 4:**
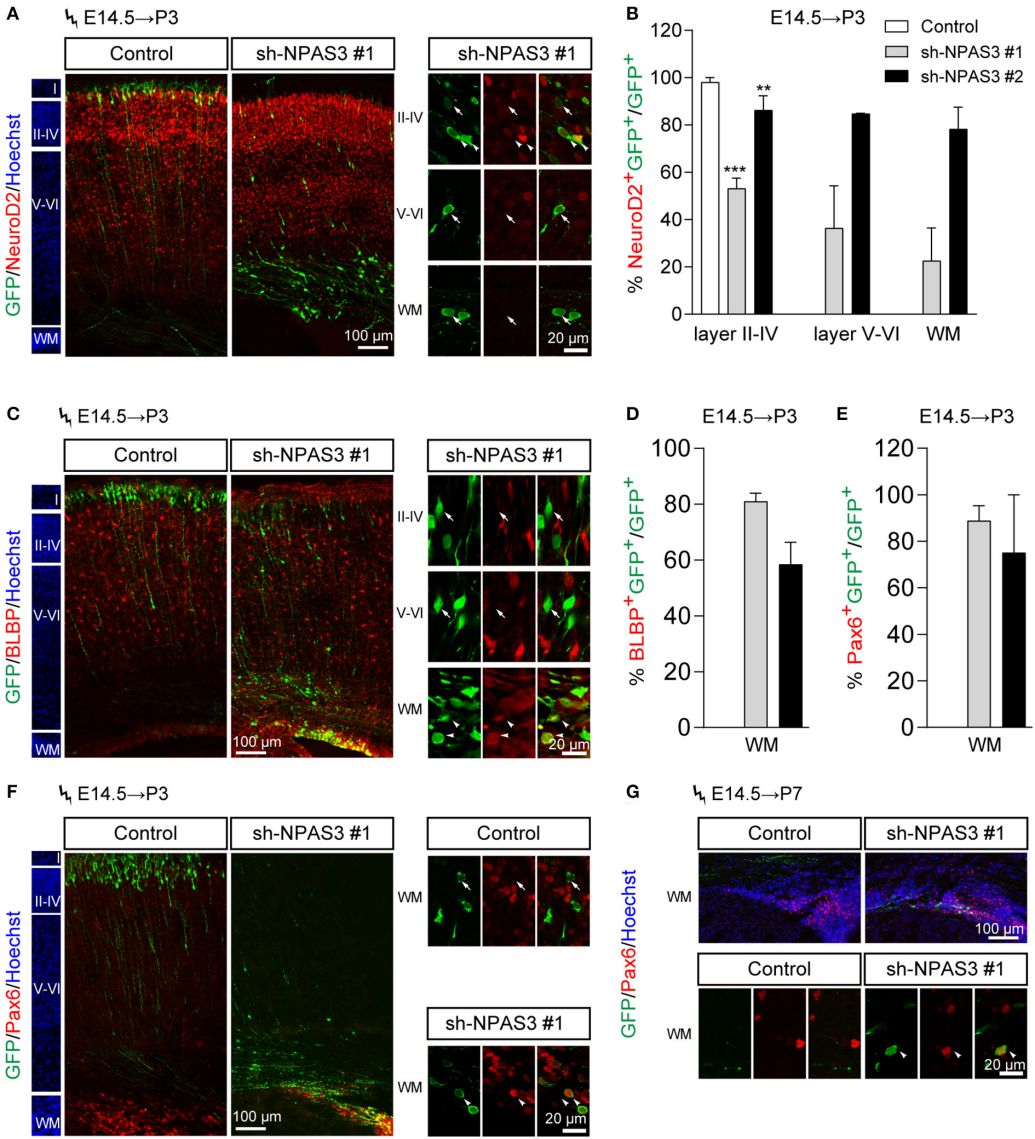
*Npas3* knockdown promoted the stemness maintenance of radial glial cells in the developing mouse cerebral cortex. *Npas3* shRNAs or pSuper-basic control plasmid together with a GFP expression plasmid were electroporated into mouse embryos at E14.5. The brains of mouse pups were harvested at P3 or P7 and processed for immunostaining with anti-GFP antibody together with antibodies against one of the following markers: NeuroD2 **(A)**, BLBP **(C)**, and Pax6 **(F, G)**. Slices were counterstained with Hoechst (blue) to indicate lamination. High-magnification images of *Npas3* knockdown cells layers II-IV, V and VI and WM were presented at the right or lower panel. Note that most *Npas3* knockdown cells in layers II-IV were Neurod2 positive (arrowheads) and BLBP negative (arrows). In contrast, the majority of *Npas3* knockdown cells in WM were NeuroD2 negative (arrows), but BLBP and Pax6 positive (arrowheads). Quantification of NeuroD2 positive cells from **(A)**, BLBP positive cells from **(C)**, and Pax6 positive cells from **(F)** are shown in **(B)**, **(D)**, and **(E)**, respectively, as % of total electroporated cells, ±SEM (*n* = 3–5). One-way ANOVA test, **p < 0.01, ***p < 0.001. WM, white matter; Cortical layers are labeled I–VI.

To further examine the cell identity of *Npas3* knockdown cells that detained in the deep cortical layers, we performed immunostaining of NeuroD2 (a pan neuron marker) to examine whether they are postmitotic neurons. It was found that most *Npas3* knockdown cells that reached layers II–IV at P3 were NeuroD2 positive, while only a proportion of *Npas3* knockdown cells that stalled in deep cortical layers were NeuroD2 positive (Figures 4A,B), indicating that some *Npas3* knockdown cells that stock in layers V–VI and white matter (WM) were not postmitotic neurons.

We further performed immunostaining of BLBP [a specific marker of radial glial cells and neonatal cortical astrocytes (Guo et al., 2009; Docampo-Seara et al., 2019)] to address if *Npas3* knockdown cells that detained in the deep cortical layers were progenitor cells. Interestingly, we found that most *Npas3* knockdown cells that stalled in WM but not layers V and VI were BLBP positive (Figures 4C,D).

Besides, we performed immunostaining of Pax6, another marker of radial glia cells, and found similar results at both P3 and P7 (Figures 4E–G). These results demonstrate that some *Npas3* knockdown cells that detained in the WM were undifferentiated radial glial cells.

**Figure 5:**
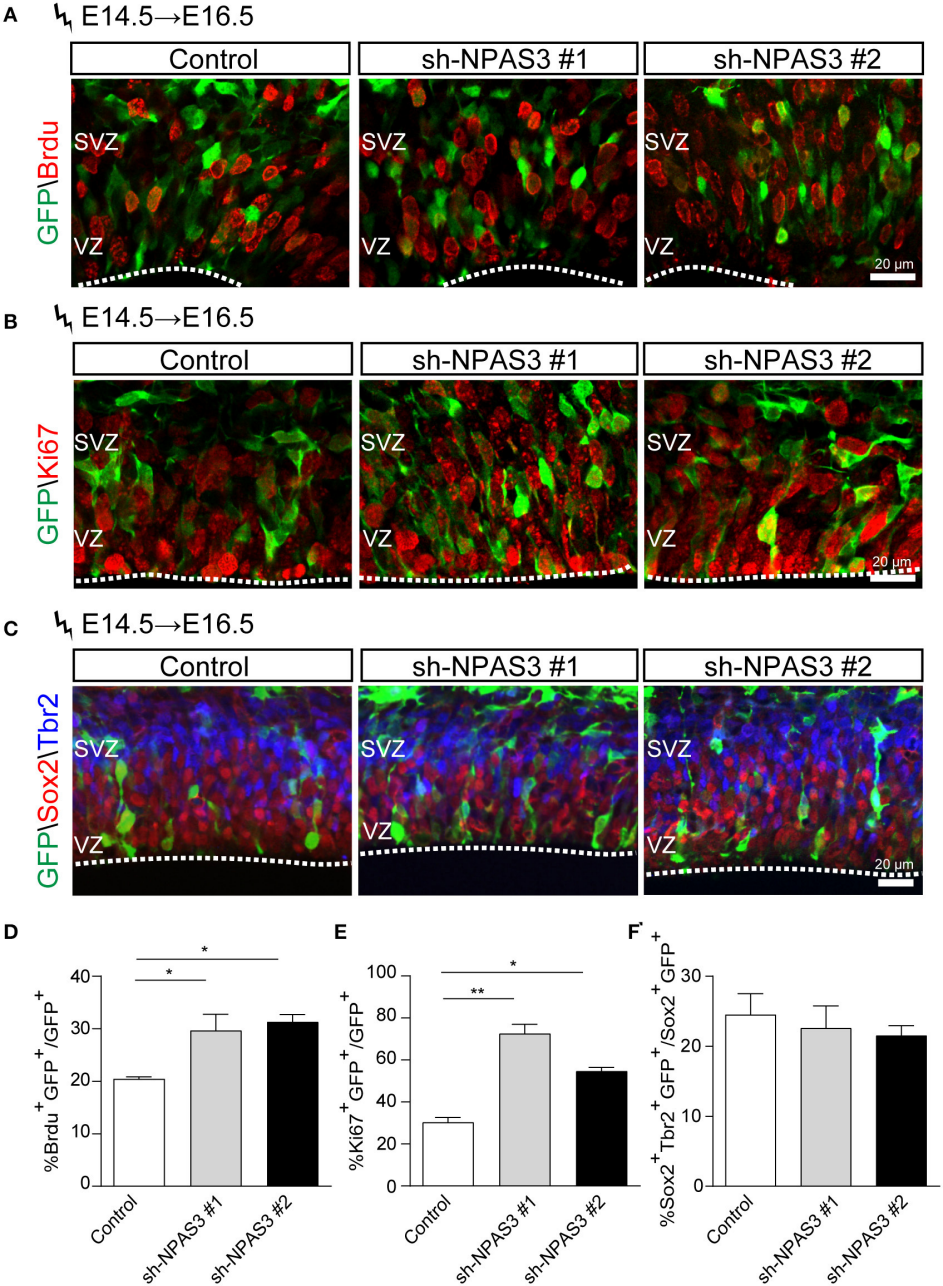
*Npas3* knockdown resulted in an increased proliferation rate of progenitors residing in VZ and SVZ. BrdU was injected into E16.5 pregnant mice 2 days after *in utero* electroporation. Mouse brains were collected 2 h later after BrdU injection, and the brain sections were processed for immunostaining with antibodies against BrdU **(A)**, Ki67 **(B)**, and Sox2/Tbr2 **(C)**. Quantification of BrdU positive cells from **(A)**, Ki67 positive cells from **(B)**, and Sox2^+^Tbr2^+^ **(C)** is shown in **(D)**, **(E)**, and **(F)**, respectively, as % of total electroporated cells, ±SEM (*n* = 3–5). The asterisks indicate the statistically significant difference with respect to control mice (one-way ANOVA test, *p < 0.05, **p < 0.01). VZ, ventricular zone; SVZ, subventricular zone.

Since most of the *Npas3* knockdown cells in the WM were radial glia cells, we performed BrdU (5-bromo-2-deoxyuridine, a thymidine analog that incorporates into dividing cells during DNA synthesis) labeling analysis (Buck et al., 2008) to investigate whether these cells show enhanced proliferation rate. We conducted *in utero* electroporation at E14.5, performed BrdU pulse labeling at E16.5, and harvested the brains 2 h after the labeling. Immunostaining of BrdU (detecting cells in S phase) and Ki67 [a classic marker for cellular proliferation (Kee et al., 2002)] revealed that *Npas3* knockdown led to an increase in the percentage of BrdU positive (control: 21.3 ± 3.4%, shRNA #1, 36.6 ± 5.4%; shRNA #2, 31.3 ± 4.0%; Figures 5A,D) and Ki67 positive cells (control: 32.0 ± 2.3%, shRNA #1, 70.0 ± 8.8%; shRNA #2, 64.5 ± 5.8%; Figures 5B,E). We further examined the radial glia to intermediate progenitor transition by colabeling Sox2 and Tbr2 (a marker of intermediate progenitor). No significant difference was observed in the proportion of Tbr2^+^Sox2^+^GFP^+^/Sox2^+^GFP^+^ cells between groups (Figures 5C,F), suggesting that *Npas3* knockdown enhances radial glia maintenance rather than affects the transition of radial glia to the intermediate progenitor.

## Discussion

*Npas3* was first identified as a candidate gene for schizophrenia through the analysis of chromosome translocation in a small family with schizophrenia. This chromosome translocation can result in either total loss of protein expression or a truncated protein with domain negative effect, both of which implicate *Npas3* downregulation. In this study, we downregulated the expression of endogenous *Npas3* by shRNAs in the progenitor cells within the cortical VZ and their progeny at E14.5 to study the role of *Npas3* in cerebral cortical development. We found that loss of *Npas3* led to neuronal radial migration defects in mice developing cerebral cortex with the impaired laminar identity of cells detained in deep cortical layers. *Npas3* deficiency also promoted radial glia stemness maintenance and enhanced proliferation rates of neural progenitor cells residing in VZ/SVZ.

There are two crucial early development stages in the formation of the cerebral cortex: the proliferation and differentiation of neural progenitor cells leading to the generation of various types of neurons, and the migration of postmitotic neurons to appropriate areas where they extend neurites and establish synaptic connections (Marin and Rubenstein, 2003). Abnormalities in these processes could lead to defects in the development of the cerebral cortex and participate in the origin of neurological disorders (Guerrini et al., 2008). Neuropathological studies have revealed the presence of cortical dysplasia (Bogerts et al., 1985; Pakkenberg, 1987) and abnormal neuronal lamination in the brain of patients with schizophrenia (Jakob and Beckmann, 1986). It is possible that impaired neuronal radial migration and laminar cell fate commitment caused by *Npas3* deficiency may contribute to the pathology of schizophrenia. During the development of the cerebral cortex, radial glial cells represented the main population of neural progenitors, dividing symmetrically and/or asymmetrically to expand the precursor population or generate postmitotic neurons (Noctor et al., 2001, 2004; Ito and Ito, 2016; Lazutkin et al., 2019), and differentiating into astrocytes after the completion of neurogenesis (Voigt, 1989; Culican et al., 1990; Hunter and Hatten, 1995; Guo et al., 2014). In this study, we found that knockdown of *Npas3* promoted the stemness maintenance of radial glial cells and increased the proliferation rates of neural progenitor cells residing in the cortical VZ/SVZ, which may further cause defects in neuronal radial migration. In consist with our results, previous studies in malignant astrocytomas have shown that loss of *NPAS3* expression was associated with the high proliferation of malignant astrocytomas in humans, while overexpression of *NPAS3* in malignant glioma cell lines significantly decreased proliferation (Moreira et al., 2011). Conversely, *Npas*3^−*/*−^ progenitors in the ganglionic eminences, the source of cortical interneurons in the developing telencephalon (Hu et al., 2017), were found to exhibit decreased proliferation and MAP kinase activity and generate fewer interneurons (Stanco et al., 2014). Besides, in human neuroblastoma SH-SY5Y cells and rat pheochromocytoma PC12 cells, cell proliferation was significantly increased following *Npas3* overexpression and decreased following *Npas3* knockdown. This discrepancy indicates that *Npas3* may play bidirectional roles in regulating cell proliferation in different types of cells. Other susceptibility genes of schizophrenia such as desmocollin 1 (*DSC1*) and neuregulin 1 (*NRG1*) had also been identified to play a vital role in regulating neuronal progenitor proliferation (Liu et al., 2005; Mao et al., 2009).

Several molecular signals have been identified to be implicated in maintaining radial glial cells, among which Notch signaling has been proposed to be a key regulator. The inactivation of Notch signaling leads to the depletion of radial glial cells and premature differentiation into intermediate progenitors and finally neurons in both embryonic and adult brains (Yoon et al., 2008; Imayoshi et al., 2010). On the contrary, the forced expression of a constitutively active form of Notch inhibits progenitors from generating neurons and keeps progenitors as proliferating radial glial cells (Mizutani and Saito, 2005). Previous studies using RNA-seq and ChIP-seq analyses identified *Notch1/2* as direct targets of *Npas3*. In addition, *Npas3* knockout mice showed a broad increase in Notch signaling as revealed by the increased expression of Notch-regulated genes such as *Stat6* and *Hes1* (Michaelson et al., 2017). We speculated that *Npas3* regulates the stemness maintenance of radial glia and proliferation rates of neural proliferation cells through modulating the activity of Notch signaling.

## Conclusion

In conclusion, here we showed that *Npas3* is critical for cortical development by influencing processes including stemness maintenance of radial glia cells, the proliferation of neural progenitors in VZ/SVZ, and neuronal radial migration, shedding light on the physiological functions of *Npas3* and increasing our understanding of the pathophysiological mechanism of *NPAS3*-related psychiatric disorders.

## Data availability statement

The raw data supporting the conclusions of this article will be made available by the authors, without undue reservation.

## Ethics statement

The animal study was reviewed and approved by All experiments involving mice were carried out in accordance with National Institute of Health Guide for the Care and Use of Laboratory Animals and approved by Animal Care and Use Committee in Shanghai Jiaotong University (IACUC No. A2019102).

## Author contributions

All authors listed have made a substantial, direct, and intellectual contribution to the work and approved it for publication.

## Funding

This study was sponsored by the Shanghai Sailing Program (Grant number 20YF1442200) and the Natural Science Foundation of Shanghai (Grant number 21ZR1455200).

## Conflict of interest

The authors declare that the research was conducted in the absence of any commercial or financial relationships that could be construed as a potential conflict of interest.

## Publisher's note

All claims expressed in this article are solely those of the authors and do not necessarily represent those of their affiliated organizations, or those of the publisher, the editors and the reviewers. Any product that may be evaluated in this article, or claim that may be made by its manufacturer, is not guaranteed or endorsed by the publisher.
