# Supplementary figures and images for: Npas3 regulates stemness maintenance of radial glial cells and neuronal migration in the developing mouse cerebral cortex
Source: Front Cell Neurosci. 2022 Oct 13;16:865681. doi: 10.3389/fncel.2022.865681 (PMC9608153; doi:10.3389/fncel.2022.865681)

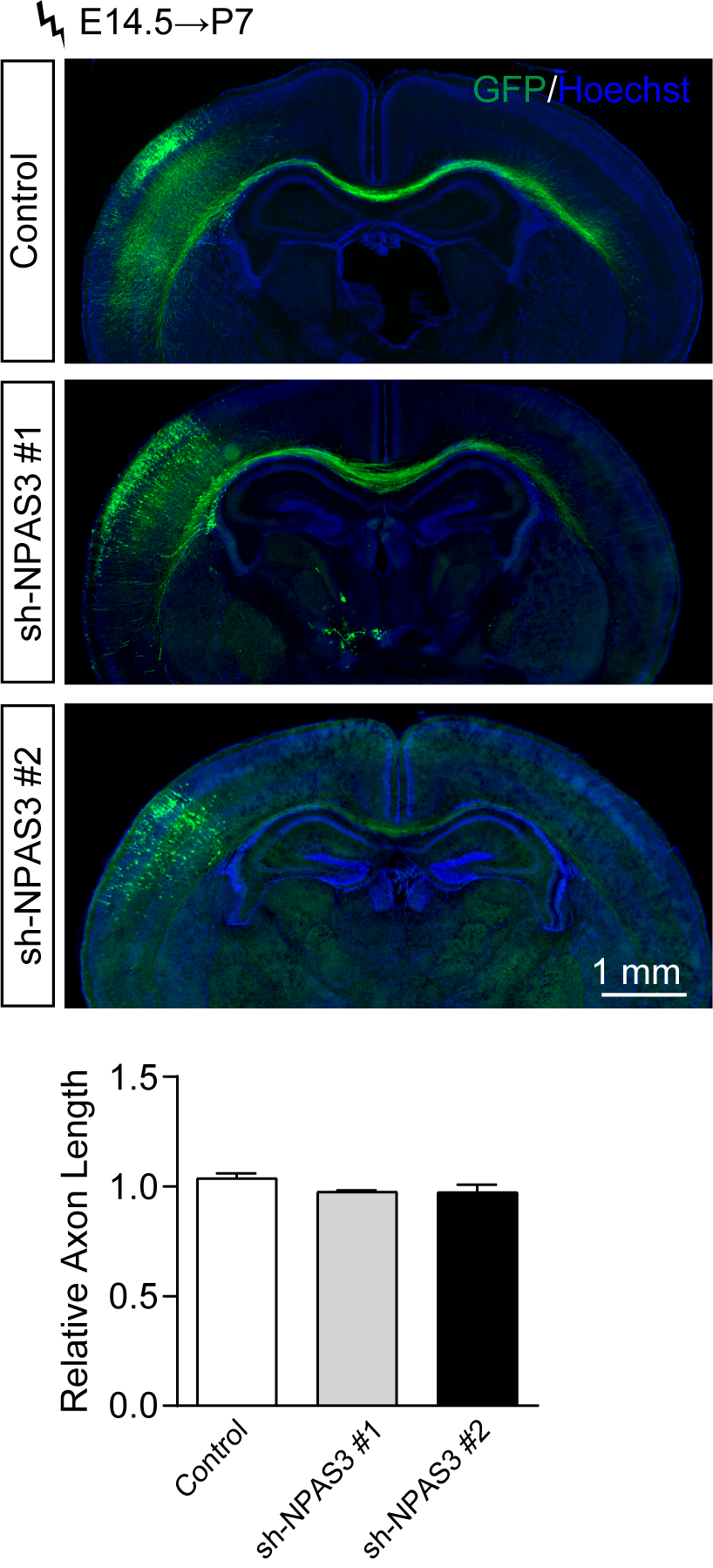

Supplement: Supplementary file 1 [file Image_1.tif]
